# Proposed intensive care unit triage models lack predictive validity for hospital mortality to deal with catastrophes: insights from a cohort study

**DOI:** 10.62675/2965-2774.20260447

**Published:** 2026-08-14

**Authors:** Paulo Marcelo Pontes Gomes de Matos, Roberta Muriel Longo Roepke, Gabriel Afonso Dutra Krelling, Larissa Bianchini, Pedro Vitale Mendes, Renato Daltro-Oliveira, Ludhmila Abrahão Hajjar, Juliana Carvalho Ferreira, Daniel Neves Forte, Leandro Utino Taniguchi, Bruno Adler Maccagnan Pinheiro Besen

**Affiliations:** 1 Universidade de São Paulo Faculdade de Medicina Hospital das Clínicas São Paulo SP Brazil Medical Intensive Care Unit, Hospital das Clínicas, Faculdade de Medicina, Universidade de São Paulo - São Paulo (SP), Brazil.; 2 Universidade de São Paulo Faculdade de Medicina Hospital das Clínicas São Paulo SP Brazil Trauma and Acute Care Surgery Intensive Care Unit, Hospital das Clínicas, Faculdade de Medicina, Universidade de São Paulo - São Paulo (SP), Brazil.; 3 A.C. Camargo Cancer Center São Paulo SP Brazil A.C. Camargo Cancer Center - São Paulo (SP), Brazil.; 4 Universidade de São Paulo Faculdade de Medicina Hospital das Clínicas São Paulo SP Brazil Division of Pulmonology, Instituto do Coração, Hospital das Clínicas, Faculdade de Medicina, Universidade de São Paulo - São Paulo (SP), Brazil.; 5 Universidade de São Paulo Faculdade de Medicina Hospital das Clínicas São Paulo SP Brazil Emergency Intensive Care Unit, Hospital das Clínicas, Faculdade de Medicina, Universidade de São Paulo - São Paulo (SP), Brazil.

**Keywords:** Ethics, Critical illness, Hospital mortality, Prognosis, COVID-19, Pandemics, Respiration, artificial, Triage, Resource allocation, Intensive care units

## Abstract

**Objective:**

To evaluate the predictive validity of the White and *Associação de Medicina Intensiva Brasileira* (AMIB) criteria in predicting hospital mortality among critically ill COVID-19 patients in Brazil.

**Methods:**

We conducted a retrospective cohort study using data from 992 mechanically ventilated COVID-19 patients admitted to a large Brazilian academic hospital, during the first pandemic wave. We applied White and AMIB triage models to this cohort and assessed their performance in predicting hospital mortality. We assessed discrimination AUROC, calibration and overall accuracy (Brier score). Decision curve analysis was used to evaluate clinical utility across varying risk thresholds.

**Results:**

Out of 992 eligible patients, the hospital mortality was 58%. The White model demonstrated moderate discrimination (AUROC 0.73; 95%CI 0.70 - 0.76), fair overall accuracy (Brier = 0.205) and adequate calibration. The AMIB model had poor discrimination (AUROC 0.67; 95%CI 0.64 - 0.70) and overall accuracy (Brier = 0.222), with poor calibration. Neither model demonstrated significant net benefit for decision thresholds above 80% risk of death.

**Conclusions:**

The White and AMIB triage models showed suboptimal performance in predicting hospital mortality in this cohort and were not effective standalone tools for intensive care unit triage, particularly for patients at the extremes of illness severity. These findings underscore the need for validation of triage tools before their implementation.

## INTRODUCTION

Triage systems are designed to guide clinicians in prioritizing care and allocating scarce resources during crises.^(1)^ Triage is most relevant following large-scale disasters such as terrorist attacks, earthquakes, or pandemics that create a surge of critically ill patients.

The coronavirus disease 2019 (COVID-19) pandemic was a major global public health crisis affecting countries across all income levels.^(2)^ Approximately 20% of hospitalized patients progressed to severe disease requiring intensive care unit (ICU) care.^(3,4)^ Despite efforts to expand capacity, demand for hospital and critical care frequently exceeded available resources.^(5–8)^

Different models have been proposed to deal with potential new threats to the healthcare system.^(9–13)^ White et al.^(14)^ suggested an allocation strategy guided by three principles: saving the most lives, saving the most life-years, and giving individuals equal opportunity to live through life's stages. Each principle is to be assessed in a four-point scale and the combined score defines each patient's priority, with higher numbers indicating a lower likelihood of benefit and, thus, a lower priority. Different societies also created guidelines orienting resource allocation during the COVID-19 threat.^(4,6,8)^ In Brazil, the *Associação de Medicina Intensiva Brasileira* (AMIB) suggested a triage tool^(15)^ based on the models by White et al.^(14)^ and Daugherty Biddison et al.^(9)^ Likewise, it is a multifaceted score-based strategy in which lower scores indicate higher priority. However, the principles and criteria used in these models were defined through expert consensus and lack validation in a real-world setting. Testing these triage tools is fundamental as dealing with bed scarcity is a daily challenge in Brazil and many other low and middle-income countries.^(8,16,17)^

We conducted this retrospective cohort study to evaluate the predictive validity of the White and AMIB criteria in predicting hospital mortality among critically ill COVID-19 patients in Brazil

## METHODS

### Study design and setting

We combined data from the COVID-19 and Frailty (CO-FRAIL) and Epidemiology of Critical COVID-19 (EPICCoV) studies to assemble a cohort of patients admitted to *Hospital das Clínicas* of the *Faculdade de Medicina* of the *Universidade de São Paulo* (USP) ICUs between March and July 2020, during the first COVID-19 wave in São Paulo, Brazil. All patients with suspected COVID-19 were included and followed for up to 60 days, with administrative censoring on July 31 or at hospital discharge, whichever occurred first.^(18)^ Both studies were approved by the *Hospital das Clínicas* of the *Faculdade de Medicina* of USP Research Ethics Committee (approval numbers 32037120.6.0000.0068 and 31382620.0.0000.0068) and registered in public registries (EPICCoV Study [Clinicaltrials.gov identifier NCT04378582] and CO-FRAIL Study [Brazilian Clinical Trials Registry identifier RBR-7w5zhr]).

During this period, the central institute of *Hospital das Clínicas* of the *Faculdade de Medicina* (USP) functioned exclusively as a referral center for severe and critical COVID-19, serving 278 secondary hospitals across 85 cities. No formal triage model was implemented, as the São Paulo State Health Department sought to provide ICU-level care to all patients requiring organ support, regardless of prognosis. Transfers occurred on a first-come, first-served basis without resource-based restrictions. Intensive care unit capacity increased from 94 pre-pandemic beds to 300 during the pandemic.^(19)^

### Participants and outcome

We included patients receiving invasive mechanical ventilation at ICU admission or intubated on the same day. During the pandemic, respiratory failure requiring invasive mechanical ventilation was the primary indication for ICU admission and the resource most at risk of rationing. We excluded patients younger than 18 years, pregnant patients, and those transferred for extracorporeal membrane oxygenation (ECMO) support, as the latter were triaged separately by a dedicated ECMO team.

### Data collection, definitions, and predictors

Data was prospectively collected during the COVID-19 pandemic as previously detailed.^(18,20)^ Data on performance status was unavailable for patients younger than 50 years old, as obtaining this information was not part of the routine of electronic health record registration at the time. We retrieved baseline and day one variables of the database, along with the hospital outcome.

We performed cross-tabulations and other cross-checks to revise database-defined variables for consistency and corrected with available data, when possible.

Age was categorized into four groups: 18 - 40 years, 41 - 60 years, 61 - 74 years, and 75 years or older, following the approach described by White et al.^(14)^

Comorbidities were defined using the Charlson Comorbidity Index and classified by projected impact on long-term survival according to White's framework as absent, minor, major, or severe. Minor comorbidities included mild cirrhosis (Child-Pugh A), asthma, hypertension, obesity, non-dialysis-dependent chronic kidney disease, and rheumatologic disease. Major comorbidities comprised dementia, malnutrition, moderate-to-severe cirrhosis (Child-Pugh B/C), chronic pulmonary disease (excluding asthma), stroke, hematologic malignancy, metastatic cancer, heart failure, AIDS, and dialysis-dependent chronic kidney disease. Severe comorbidity was defined as any major condition combined with frailty (Clinical Frailty Scale [CFS] ≥ 5).^(21)^

The Sequential Organ Failure Assessment (SOFA) score was categorized according to two different frameworks. For the AMIB criteria, SOFA was divided into four categories: zero to 8, 9 - 11, 12, 13, and 14 - 24. For White's criteria, categories were defined as zero to 5, 6 - 9, 10 - 12, and 13 - 24.

Functional status was assessed using the Eastern Cooperative Oncology Group (ECOG) performance status, which was derived from the Karnofsky Performance Status (KPS) as follows:^(22)^ KPS of 100% corresponded to ECOG 0; KPS of 80 - 90% to ECOG 1; KPS of 50 - 70% to ECOG 2; and KPS of 40% or lower to ECOG 3. Patients with very severe frailty (CFS ≥ 8) were classified as ECOG 4, reflecting a condition of extreme debilitation or moribund status. Patients younger than 50 years old were assigned an ECOG of 0 for the primary analysis.

To derive the AMIB discrete categories, ECOG scores of zero to one were assigned a weight of one, with each increase in ECOG category adding one point. Major comorbidities were weighted three. Similarly, each incremental increase in SOFA categories contributed 1 point, with a total score ranging from 2 to 11 points.

To derive the White scoring system, each SOFA, age, and comorbidities category had an increasing weight of 1, with total scores ranging from 3 to 12 points.

We then derived, with the available data, the AMIB triaging scoring system,^(15)^ which was proposed early in the pandemic, and the White triaging scoring system.^(14)^ We selected these models because the former was recommended in Brazil as a triaging tool, while the latter has good acceptance in the literature and provides the theoretical framework for other systems.^(9)^

### Data analysis

We stratified the descriptive analysis according to hospital outcome (mortality). We present means and standard deviations (SD) or medians and 25^th^/75^th^ percentiles [P25 - P75], as appropriate for continuous variables, with t-tests and Wilcoxon rank-sum tests for comparison respectively. For categorical variables, we present absolute counts and proportions with Fisher exact tests for comparisons.

We assessed the predictive validity of the AMIB and White models for hospital mortality on the entire cohort as our primary analysis. We also performed a sensitivity analysis excluding patients younger than 50 years for whom performance status data was unavailable. As an exploratory analysis, we evaluated the contribution of each individual component to the overall predictive accuracy of the models. To do so, we compared the performance of each complete model against its individual components, including age categories, the presence of severe comorbidities, discrete SOFA categories, discrete ECOG categories, and of a model including all variables in their best functional format (splines for age, linear for SOFA, categories for ECOG and comorbidities).

For each model, we compared their apparent validity within this dataset, with discrimination (area under the receiving operator characteristic curves [AUROC]), calibration (Hosmer-Lemeshow goodness-of-fit test and calibration plots), the Brier score, precision-recall curve analysis and net benefit analyses through decision curve analysis (DCA) plots.

The Brier score is a metric that assesses the precision of probabilistic predictions for binary outcomes. Unlike metrics that focus solely on discrimination (e.g., AUROC), the Brier Score assesses overall predictive accuracy by measuring the mean squared difference between the predicted probabilities and the actual observed outcomes. The score ranges from zero to one, in which zero represents perfect accuracy and one indicates total inaccuracy. For binary outcomes, a score below 0.25 is generally required to outperform a non-informative model.^(23)^

The DCA plot presents the net benefit of adopting each different model across various threshold probabilities of the outcome (hospital mortality) to guide individual decisions (ICU triage). Importantly, there is no definite predicted mortality threshold for ICU admission. The point of intersection between the "Treat all" and the "Treat none" strategies, represents the cohort observed hospital mortality. We considered patients with expected hospital mortality greater than 80% would be fit for ICU triage.

For AUROC, we present asymptotic 95% confidence intervals, without pairwise comparisons. For the Brier's score, bootstrap (n = 200) confidence intervals were obtained. We performed an additional post-hoc sensitivity analysis for the missing data for participants below 50 years of age using multiple imputation, detailed in the supplemental file. For plot generation, we used the user-written Stata SE pmcalplot, dca and prtab commands. All analyses were done in Stata SE 18.0, with p-values presented to the nominal 0.05 value.

## RESULTS

### Sample characteristics

**Figure 1:**
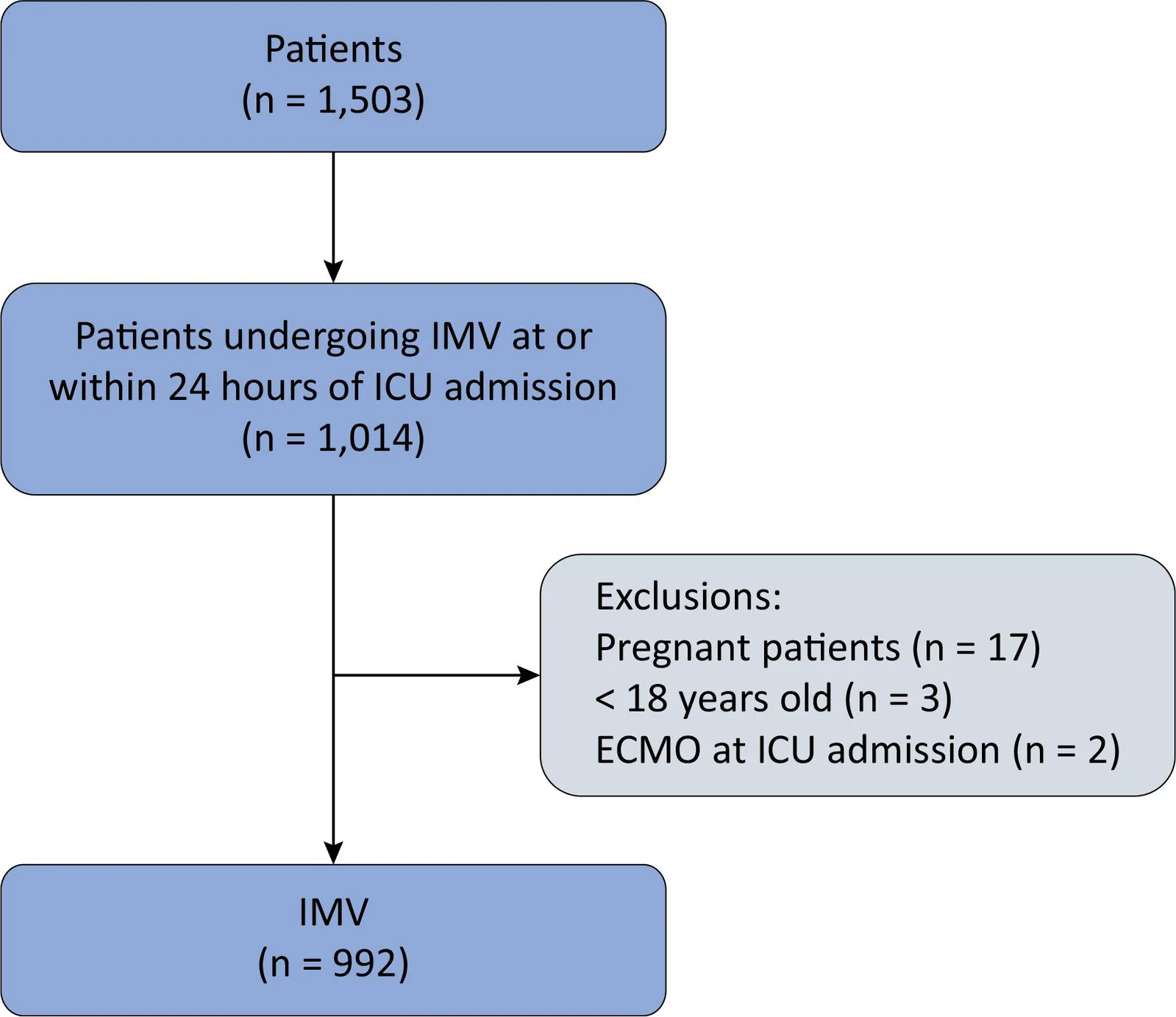
Study participants.

Between March 2020 and July 2020, 1,503 patients were assessed for eligibility, of which 1,014 were either undergoing IMV or were intubated within 24h of ICU admission (Figure 1). Seventeen patients were pregnant, three were younger than 18 years old and 2 were undergoing ECMO at ICU admission, leaving a final cohort of 992 patients, with 577 (58%) deceased patients.

IMV - invasive mechanical ventilation; ICU - intensive care unit; ECMO - extracorporeal membrane oxygenation.

The mean ± SD age was 60.7 ± 14 years and 60.7% were male. Arterial hypertension was the most common comorbidity in the overall cohort (58.7%), followed by obesity (26.2%). Heart failure was the most common major comorbidity (11.2%) (Table 1S - Supplementary Material). Overall, 136 patients (13.7%) were classified as frail (CFS ≥ 5) (Table 1).

**Table 1:** Sample characteristics stratified by hospital mortality

Variables		Survivors (n = 415)	Deceased (n = 577)	p value
Age		55.4 ± 13.4	64.6 ± 13.2	< 0.001
Age categories (years)				< 0.001
	18 - 40	57 (13.7)	29 (5.0)	
	41 - 60	198 (47.7)	168 (29.1)	
	61 - 74	133 (32.0)	253 (43.8)	
	≥ 75	27 (6.5)	127 (22.0)	
Male sex		236 (56.9)	366 (63.4)	0.041
Minor comorbidities		233 (56.1)	265 (45.9)	0.002
Major comorbidities		68 (16.4)	155 (26.9)	< 0.001
Severe comorbidities		11 (2.7)	59 (10.2)	< 0.001
Clinical Frailty Scale				< 0.001
	1	182 (43.9)	160 (27.7)	
	2	56 (13.5)	69 (12.0)	
	3	111 (26.7)	168 (29.1)	
	4	35 (8.4)	75 (13.0)	
	5	16 (3.9)	51 (8.8)	
	6	10 (2.4)	34 (5.9)	
	7	4 (1.0)	17 (2.9)	
	8	1 (0.2)	3 (0.5)	
ECOG categories				< 0.001
	0/1	378 (91.1)	465 (80.6)	
	2	28 (6.7)	80 (13.9)	
	3	8 (1.9)	29 (5.0)	
	4	1 (0.2)	3 (0.5)	
AMIB score		2 [2 - 3]	3 [2 - 5]	< 0.001
White score		6 [5 - 7]	7 [6 - 8]	< 0.001
Timing of invasive mechanical ventilation				0.055
At ICU admission		382 (92.0)	509 (88.2)	
Within 24h of ICU admission		33 (8.0)	68 (11.8)	
Vasoactive drugs use		178 (42.9)	360 (62.4)	< 0.001
Renal replacement therapy		15 (3.6)	75 (13.0)	< 0.001
SOFA score		6 [4 - 8]	8 [6 - 10]	< 0.001
SOFA components				
	Respiratory	3 [2 - 3]	3 [2 - 3]	< 0.001
	Hepatic	0 [0 - 0]	0 [0 - 0]	0.65
	Neurologic	1 [0 - 1]	1 [0 - 1]	0.15
	Cardiovascular	0 [0 - 3]	3 [0 - 4]	< 0.001
	Hematological	0 [0 - 0]	0 [0 - 0]	< 0.001
	Renal	0 [0 - 2]	2 [0 - 3]	< 0.001

ECOG - Eastern Cooperative Oncology Group; AMIB - *Associação de Medicina Intensiva Brasileira*; ICU - intensive care unit; SOFA - Sequential Organ Failure Assessment. Results expressed as mean ± standard deviation, n (%) or median [interquartile range].

Deceased patients were significantly older (64.5 ± 13.2 *versus* 55.4 ± 13.4 years); had higher prevalence of major or severe comorbidities (37.1% *versus* 19.1%); and higher median [P25 - P75] SOFA score (8 [6; 10] *versus* 6 [4; 8]) than survivors (Table 1). Timing of initiation of mechanical ventilation was equivalent in both groups, but the deceased had a higher use of vasoactive drugs (62.4% *versus* 42.9%; p < 0.001) and renal replacement therapy (13% *versus* 3.6%; p < 0.001) at baseline (Table 1).

### Main results

**Figure 2:**
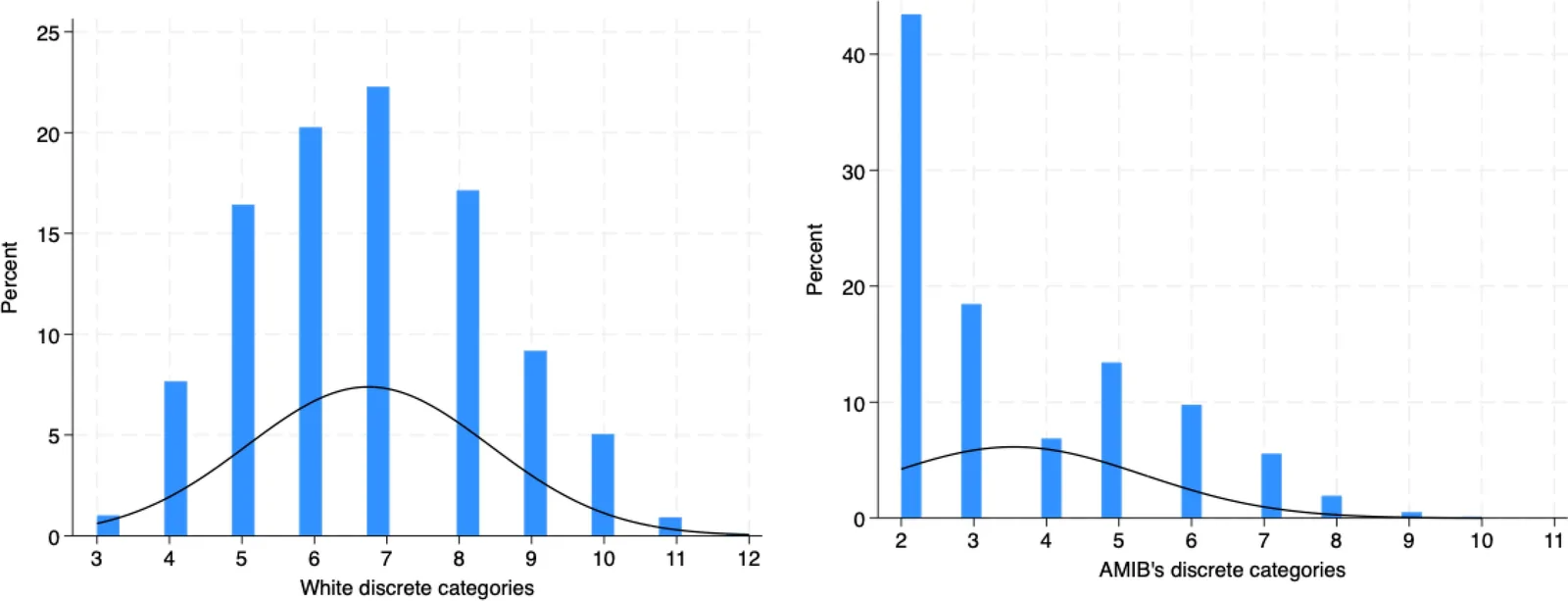
Distribution of White's and AMIB's criteria discrete categories.

White's model distributed patients more evenly across intermediate categories, following a normal distribution, while AMIB's model concentrated patients in the lower categories (Figure 2).

AMIB - *Associação de Medicina Intensiva Brasileira.*

**Figure 3:**
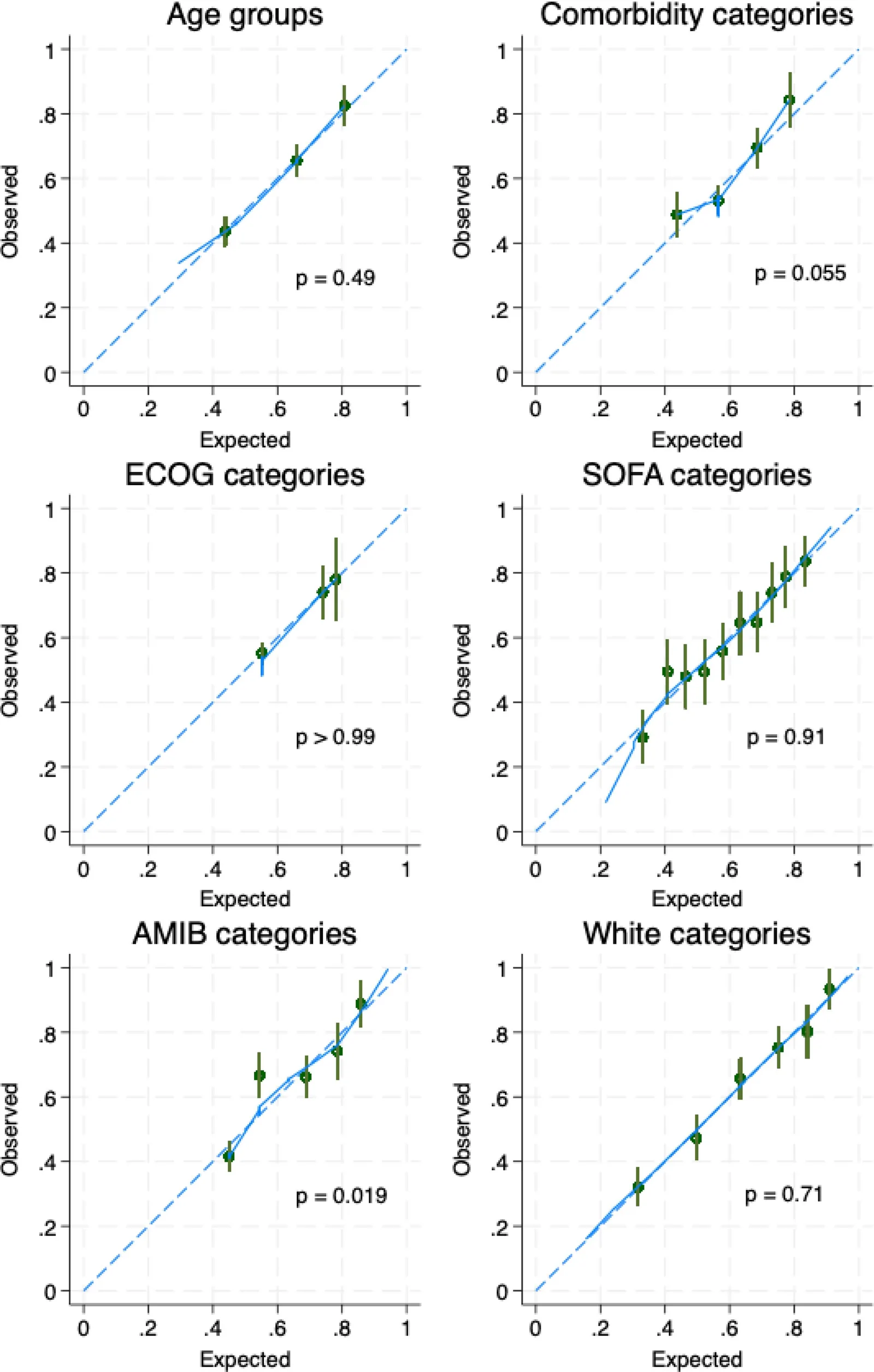
Calibration plots.

White's model had a better overall performance for predicting hospital mortality when compared to AMIB's model (AUROC 0.73 *versus* 0.67, Table 2). This is supported by the analysis of the precision-recall curve (Figure 1S - Supplementary Material) which demonstrates a better positive predictive value for White's model along the sensitivity thresholds. Also, White's model showed adequate apparent calibration, while AMIB's model was poorly calibrated (Figure 3).

ECOG - Eastern Cooperative Oncology Group; SOFA - Sequential Organ Failure Assessment; AMIB - *Associação de Medicina Intensiva Brasileira*.

These calibration plots demonstrate White's criteria and AMIB's criteria LOWESS (Locally Weighted Scatterplot Smoothing) curves (blue lines), along with their discrete categories with 95% confidence intervals (green). Age groups, comorbidities, Eastern Cooperative Oncology Group and Sequential Organ Failure Assessment categories are also presented independently as they are components of either White's or AMIB's criteria.

**Table 2:** Discrimination, accuracy (Brier score) and calibration of the proposed models

Model	AUROC (95%Cis*)	Brier score (95%CIs†)	HL GOF‡
Age	0.67 (0.63 - 0.70)	0.222 (0.212 - 0.231)	0.49
Major comorbidity	0.61 (0.57 - 0.64)	0.234 (0.223 - 0.241)	0.05
SOFA categories	0.68 (0.65 - 0.71)	0.219 (0.209 - 0.229)	0.91
ECOG categories	0.55 (0.53 - 0.57)	0.24 (0.23 - 0.24)	‡
AMIB criteria	0.67 (0.64 - 0.70)	0.222 (0.214 - 0.232)	< 0.001
White criteria	0.73 (0.70 - 0.76)	0.205 (0.194 - 0.216)	0.70

AUROC - area under the receiver operating characteristic curve; HL GOF - Hosmer-Lemeshow goodness-of-fit test; SOFA - Sequential Organ Failure Assessment; ECOG - Eastern Cooperative Oncology Group; AMIB - *Associação de Medicina Intensiva Brasileira.*

* Asymptotic 95% confidence intervals;

† Bootstrapped 95% confidence intervals (n = 200);

‡ Goodness of fit test cannot be carried due to zero degrees of freedom.

**Figure 4:**
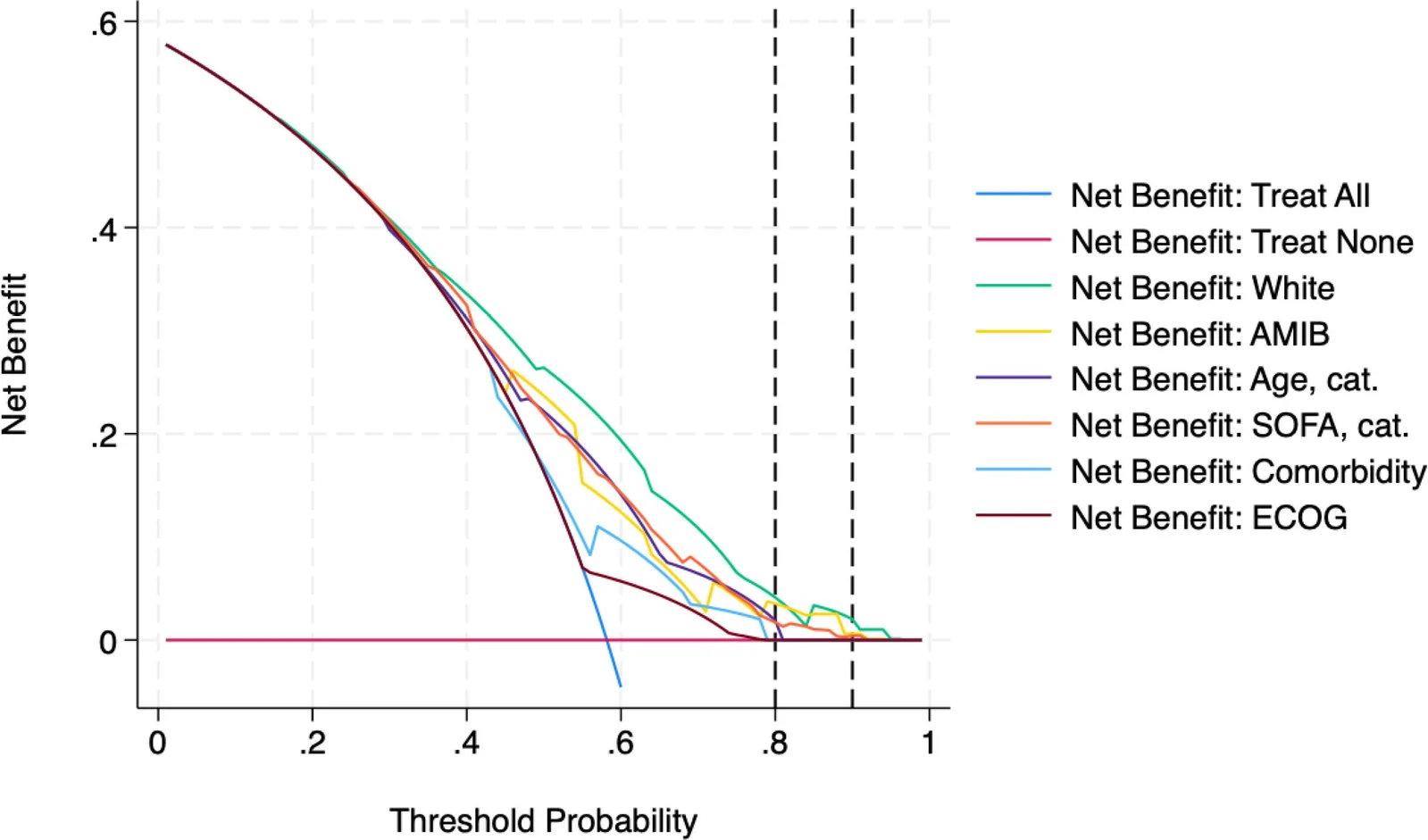
Decision curve analysis plots.

In a DCA plot (Figure 4) White's model had the highest net benefit across intermediate threshold probabilities of 40 - 80%. No model demonstrated meaningful benefit in decision thresholds greater than 80%.

AMIB - *Associação de Medicina Intensiva Brasileira*; SOFA - Sequential Organ Failure Assessment; ECOG - Eastern Cooperative Oncology Group.

The decision curve analysis plot presents the net benefit of adopting each different model across threshold probabilities of hospital mortality to make individual decisions (i.e., in this case, intensive care unit triage). The x-axis points where the "Treat all" strategy meets the "Treat none" strategy represents the cohort observed hospital mortality. The vertical dashed lines represent 80% and 90% threshold probabilities, which could be considered as thresholds to identify patients less suitable to benefit from intensive care unit care. This figure demonstrates that, although the White model has higher net benefit across intermediate threshold probabilities (where no decision-making is likely to be made upon), no model has meaningful net benefit beyond 80% or 90% thresholds.

### Sensitivity and secondary analyses

The sensitivity analysis within the cohort of patients over 50 years old for whom performance status was available consisted of 684 patients (Tables 2S and 3S - Supplementary Material). The distribution of the score was similar to the main analysis (Figure 2S - Supplementary Material). AMIB and White's models displayed similar calibration (Figure 3S - Supplementary Material) and DCA plots (Figure 4S - Supplementary Material) when compared to the full cohort.

The secondary analyses of assessment of each variable's predictive performance showed that SOFA categories and age had a similar performance to the AMIB model, but with better calibration (Table 2 and Figures 3 and 4). Models based on the presence of major comorbidities or ECOG alone performed poorly with limited discrimination, accuracy, and inadequate calibration. These results were also similar in the cohort of patients older than 50 years-old (Table 4S - Supplementary Material).

The model with all variables in their best functional format (splines for age, linear function for SOFA, categories for ECOG and comorbidities) demonstrated the best fit in the comparison of logistic regression models with a Bayesian Information Criterion (BIC) of 1211.3 compared to 1233.5 and 1290.6 for White and AMIB models, respectively (Table 5S - Supplementary Material). These results were also similar in the cohort of patients older than 50 years-old (Table 6S - Supplementary Material).

The post-hoc sensitivity analysis with imputation of missing data for participants below 50 years of age yielded similar results (Table 7S, Figures 5S and 6S - Supplementary Material).

## DISCUSSION

In this retrospective cohort of patients admitted to the ICU during the first wave of the COVID-19 pandemic, we observed that White's model had a fair performance for discriminating survivors and non-survivors with better differentiation across the full spectrum of categories when compared to the AMIB model. However, none of the models were suitable for decision making in the extremes of probability thresholds.

The exact threshold of when intensive care becomes potentially inappropriate is unknown as some clinicians would still be keen to admit a patient with a lower than 20% likelihood of survival. At the same time, even a patient with an estimated mortality of less than 40% might still be unsuitable for the ward, especially if the expected disease course is of continued deterioration. Preventing these patients from being admitted to the ICU may harm their clinical course, as part of the goal of intensive care is to act swiftly to prevent further deterioration. Triage is about making difficult decisions and it can be argued that the major role of an ICU triage model is to adequately identify groups that lie in the extremes of mortality probabilities.^(24,25)^ When looking at patients with a lower likelihood of benefit from ICU care – those with an expected mortality inferior to 20% or greater than 80% – we found the models were not superior to "treat all" or "treat none" approaches.

In the DCA plot, White's model provided the highest net benefit around threshold probabilities of 40 - 80%. The AMIB model had a worse overall performance – similar to age alone, which is an inappropriate triaging tool^(24,26–28)^ – and was poorly calibrated. These characteristics suggest this model is unsuitable for utilization. However, it may have been penalized in the main analysis, as functional status parameters were unavailable for patients younger than 50 years-old, for whom we assumed a good performance status. We addressed this limitation by conducting a sensitivity analysis among patients over 50 years old which yielded similar conclusions.

Others have also observed that triaging patients based on age, severe comorbidities, and SOFA score^(29)^ or using an ethical triaging tool^(30)^ would lead to increased bed-availability but at the cost of failing to admit many patients with considerable short and long-term survival. Additionally, we observed that the best discriminating model would be one that includes variables avoiding categorization of age and SOFA, which leads to unnecessary loss of discriminating capacity.

Our results are in line with the long-standing recommendation that illness severity scores should not be used for clinical decision making. In a large cohort, Shahpori et al.^(31)^ demonstrated significant outcome heterogeneity at a fixed SOFA score of 11, with mortality rates ranging from 29% to 67% depending on the diagnostic category. Age had a similar impact. These findings underscore that a static SOFA threshold is an imperfect determinant of survival.

In fact, these scores were not designed to assist clinicians making individual patient decisions^(32–34)^ and their performance may be worse than a trained physician's assessment.^(35–37)^ However, caution is advised when using subjective criteria as they may be biased.^(38,39)^ Including a correction factor to reduce structural inequities or prioritization of high-risk essential workers,^(40)^ using a structured framework to guide decision making^(41,42)^ and triaging by a clinician not involved in patient care^(6,24,25,28)^ are possible mitigating solutions.

These results imply the need for further research, including development and validation of catastrophe triage models which combine ethical concepts with adequate discrimination, calibration and decision analysis properties in real-world settings.

### Strengths and limitations

Our study's main strength is the use of a data-driven approach to validation of the proposed models, with the incorporation of modern methods for decision analysis, such as the DCA. We also had a reasonable sample size to derive precise estimates of accuracy for this validation. Finally, it was validated in a middle-income scenario, where scarcity of resources is a present reality.

This study has many limitations. First, we conditioned our analysis on patients at the time of ICU admission. Illness severity scores then could differ from those calculated at the point when triage was expected to happen. However, during the first wave of the pandemic, there was no explicit triaging, but implicit triaging on a first-come first-served basis, where all patients in need of mechanical ventilation were admitted to available ICU beds. Nevertheless, it is possible that some patients could have been exposed to hidden triage before ICU admission. Second, frailty is a strong predictor of both short- and long-term outcomes in critically ill patients^(42–45)^ – independently from age^(46)^ – and our data differs from the models proposed by AMIB and White due to missing data regarding functional status and severe comorbidities as defined by the authors. However, other publications have derived ECOG from KPS^(22,47)^ and on a sensitivity analysis including only patients for whom this information was available our results remained the same. Third, these data are from a single large academic health center and may not represent the Brazilian ICU population. The mortality rate observed in our study was lower than the national average (80%) reported during the first wave of the pandemic.^(48)^ This may reflect selection bias, as our facility serves as a reference center for high complexity critically ill patients and may have been better prepared for the pandemic surge.

## CONCLUSION

The predictive validity of two proposed models for intensive care unit triage during the COVID-19 pandemic was suboptimal in this validation study. Although our data has many limitations, these results suggest that further efforts for data-driven validation of such models are necessary before wide implementation, especially in the context of a pandemic, where decision-making uncertainty is high.

## Data Availability

Data is available on demand from referees.
