# Supplementary material for: Proposed intensive care unit triage models lack predictive validity for hospital mortality to deal with catastrophes: insights from a cohort study
Source: Crit Care Sci. 2026 Aug 14;38:e20260447. doi: 10.62675/2965-2774.20260447 (PMC13518022; doi:10.62675/2965-2774.20260447)
Supplement: Supplementary file 1 [file 2965-2774-ccsci-38-e20260447-Suppl01.pdf]

Critical Care Science
https://doi.org/10.62675/2965-2774.20260447

SUPPLEMENTARY MATERIAL

Proposed intensive care unit triage models lack predictive validity for hospital mortality to deal with catastrophes: insights from a cohort study

Paulo Marcelo Pontes Gomes de Matos[1], Roberta Muriel Longo Roepke[2], Gabriel Afonso Dutra Krelling[1], Larissa Bianchini[1], Pedro Vitale Mendes[1], Renato Daltro-Oliveira[3], Ludhmila Abrahão Hajjar[1], Juliana Carvalho Ferreira[4], Daniel Neves Forte[5], Leandro Utino Taniguchi[1], Bruno Adler Maccagnan Pinheiro Besen[1]

Table 1S - Comorbidities stratified by hospital mortality

Variables	Total	Survivors	Deceased	p value
n	992	415	577	
Dementia	9 (0.9)	1 (0.2)	8 (1.4)	0.088
Malnutrition	7 (0.7)	2 (0.5)	5 (0.9)	0.71
Chronic pulmonary disease - not asthma	58 (5.8)	17 (4.1)	41 (7.1)	0.054
Moderate or Severe Cirrhosis	10 (1.0)	3 (0.7)	7 (1.2)	0.53
Chronic hematological disorder	4 (0.4)	1 (0.2)	3 (0.5%)	0.64
Previous TIA or stroke	37 (3.7)	10 (2.4)	27 (4.7)	0.088
Chronic heart disease	111 (11.2)	31 (7.5)	80 (13.9)	0.002
HIV/AIDS	16 (1.6)	3 (0.7)	13 (2.3)	0.074
CKD - dialysis	23 (2.3)	9 (2.2)	14 (2.4)	0.83
Cancer	77 (7.8)	14 (3.4)	63 (10.9)	< 0.001
Mild chronic hepatic disease	5 (0.5)	2 (0.5)	3 (0.5)	1.00
Diabetes mellitus - no complications	84 (8.5)	22 (5.3)	62 (10.7)	0.003
Asthma	29 (2.9)	11 (2.7)	18 (3.1)	0.71
Essential hypertension	582 (58.7)	223 (53.7)	359 (62.2)	0.009
Obesity	260 (26.2)	132 (31.8)	128 (22.2)	< 0.001
CKD - non-dialysis	57 (5.7)	8 (1.9)	49 (8.5)	< 0.001
Rheumatologic disease	17 (1.7)	7 (1.7)	10 (1.7)	1.00

TIA - transient ischaemic attack; CKD - chronic kidney disease. Results expressed as n (%), when not expressed differently.

This is an open access article under the CC BY license (https://creativecommons.org/licenses/by/4.0/).

Table 2S - Sample characteristics stratified by hospital mortality for patients over 50 years old

	Survivors (n = 249)	Deceased (n = 435)	p value
Age	63.3 ± 8.1	68.2 ± 9.6	< 0.001
Age categories (years)			< 0.001
51 - 60	103 (41.4)	105 (24.1)	
61 - 74	123 (49.4)	217 (49.9)	
≥ 75	23 (9.2)	113 (26.0)	
Male sex	116 (46.6)	154 (35.4)	0.004
Minor comorbidities	148 (59.4)	210 (48.3)	0.005
Major comorbidities	42 (16.9)	96 (22.1)	0.11
Severe comorbidities	11 (4.4)	59 (13.6)	< 0.001
Clinical Frailty Scale			0.009
1	16 (6.4)	18 (4.1)	
2	56 (22.5)	69 (15.9)	
3	111 (44.6)	168 (38.6)	
4	35 (14.1)	75 (17.2)	
5	16 (6.4)	51 (11.7)	
6	10 (4.0)	34 (7.8)	
7	4 (1.6)	17 (3.9)	
8	1 (0.4)	3 (0.7)	
ECOG categories AMIB			0.007
1	212 (85.1)	323 (74.3)	
2	28 (11.2)	80 (18.4)	
3	8 (3.2)	29 (6.7)	
4	1 (0.4)	3 (0.7)	
AMIB score	2 [2 - 4]	3 [2 - 6]	< 0.001
White score	6 [5 - 7]	7 [6 - 8]	< 0.001
Timing of invasive mechanical ventilation			
At ICU admission	231 (92.8)	385 (88.5)	
Within 24h of ICU admission	18 (7.2)	50 (11.5)	
Vasoactive drugs use	114 (45.8)	273 (62.8)	< 0.001
Renal replacement therapy	7 (2.8)	57 (13.1)	< 0.001
SOFA score	6 [4 - 8]	8 [6 -10]	< 0.001
SOFA components			
Respiratory	3 [2 - 3]	3 [2 - 3]	0.003
Hepatic	0 [0 - 0]	0 [0 - 0]	0.68
Neurologic	1 [0 - 1]	1 [0 - 1]	0.33
Cardiovascular	1 [0 - 3]	3 [0 - 4]	< 0.001
Hematological	0 [0 - 0]	0 [0 - 0]	< 0.001
Renal	1 [0 - 2]	2 [0 - 3]	< 0.001

ECOG - Eastern Cooperative Oncology Group; AMIB - *Associação de Medicina Intensiva Brasileira*; ICU - intensive care unit; SOFA - Sequential Organ Failure Assessment. Results expressed as mean ± standard deviation, n (%) or median (interquartile range).

Table 3S - Comorbidities stratified by hospital mortality in patients over 50 years old

Variables	Survivors	Deceased	p value
n	249	435	
Dementia	0	8 (1.8)	0.06
Malnutrition	1 (0.4)	3 (0.7)	1.00
Chronic pulmonary disease - not asthma	15 (6.0)	32 (7.4)	0.53
Moderate or severe cirrhosis	1 (0.4)	5 (1.1)	0.42
Chronic hematological disorder	0	2 (0.5)	0.54
Previous TIA or stroke	6 (2.4)	22 (5.1)	0.11
Chronic heart disease	23 (9.2)	61 (14)	0.07
HIV/AIDS	0	8 (1.8)	0.06
CKD - dialysis	5 (2)	9 (2.1)	1.00
Cancer	12 (4.8)	44 (10.1)	0.01
Mild chronic hepatic disease	2 (0.8)	2 (0.5)	0.62
Diabetes mellitus - no complications	17 (6.8)	51 (11.7)	0.05
Asthma	4 (1.6)	12 (2.8)	0.44
Essential hypertension	172 (69.1)	289 (66.4)	0.50
Obesity	67 (26.9)	84 (19.3)	0.03
CKD - non-dialysis	8 (3.2)	40 (9.2)	< 0.01
Rheumatologic disease	5 (2)	7 (1.6)	0.77

TIA - transient ischaemic attack; CKD - chronic kidney disease. Results expressed as n (%), when not expressed differently.

Table 4S - Discrimination, accuracy (Brier score), and calibration of the proposed models among patients older than 50 years

Model	AUROC (95%Cis*)	Brier score (95%CIs†)	HLGOF
Age	0.63 (0.59 - 0.67)	0.219 (0.207 - 0.231)	0.17
Major comorbidity	0.58 (0.54 - 0.62)	0.227 (0.217 - 0.236)	0.06
SOFA categories	0.67 (0.63 - 0.71)	0.211 (0.199 - 0.223)	0.90
ECOG categories	0.56 (0.52 - 0.59)	0.228 (0.218 - 0.238)	‡
AMIB criteria	0.65 (0.61 - 0.69)	0.218 (0.207 - 0.229)	0.018
White criteria	0.70 (0.66 - 0.74)	0.206 (0.193 - 0.218)	0.54

AUROC - area under the Receiver Operating Characteristic curve; HLGOF - Hosmer-Lemeshow goodness-of-fit test; SOFA - Sequential Organ Failure Assessment; ECOG - Eastern Cooperative Oncology Group; AMIB - *Associação de Medicina Intensiva Brasileira*. * Asymptotic 95% confidence intervals; † Bootstrapped 95% confidence intervals (n = 200); ‡ Goodness of fit test cannot be carried out due to zero degrees of freedom.

Table 5S - Logistic regression model results of the White score and AMIB score compared to a more flexible score incorporating all variables

Variable	Model 1 (Spline age + SOFA continuous + White + ECOG AMIB) OR (95%CI)	Model 2 (White score) OR (95%CI)	Model 3 (AMIB Score) OR (95%CI)
Non-linear spline function of age	Spline ($p < 0.001$)	NA	NA
Age (years)			
18 - 40	NA	Reference	NA
41 - 60	NA	1.8 (1.1 - 3.1); $p = 0.023$	NA
61 - 74	NA	3.3 (1.9 - 5.6); $p < 0.001$	NA
≥ 75	NA	8.3 (4.4 - 15.9); $p < 0.001$	NA
SOFA			
1 point	1.2 (1.2 - 1.3); $p < 0.001$	NA	NA
1st level (AMIB 0 - 8; White 0 - 5)	NA	Reference	Reference
2nd level (AMIB 9 - 11; White 6 - 9)	NA	1.87 (1.4 - 2.6); $p < 0.001$	2.45 (1.8 - 3.4); $p < 0.001$
3rd level (AMIB 12 - 13; White 10 - 12)	NA	3.92 (2.6 - 5.9); $p < 0.001$	4.41 (2.4 - 8.1); $p < 0.001$
4th level (AMIB 14 - 24; White 13 - 24)	NA	11.5 (3.9 - 33.9); $p < 0.001$	13.9 (1.8 - 106.6); $p = 0.011$
Comorbidities			
Minor	0.84 (0.6 - 1.2); $p = 0.346$	0.96 (0.7 - 1.4); $p = 0.817$	NA
Major	1.45 (0.9 - 2.3); $p = 0.098$	1.66 (1.1 - 2.6); $p = 0.022$	2.05 (1.5 - 2.8); $p < 0.001$
Severe	2.22 (0.9 - 5.2); $p = 0.067$	2.97 (1.4 - 6.3); $p = 0.004$	NA
ECOG categories			
0 - 1	Reference	NA	Reference
2	1.01 (0.6 - 1.8); $p = 0.981$	NA	1.99 (1.2 - 3.2); $p = 0.004$
3	1.47 (0.6 - 3.8); $p = 0.418$	NA	2.47 (1.1 - 5.6); $p = 0.031$
4	0.43 (0 - 5); $p = 0.5$	NA	1.26 (0.1 - 13.2); $p = 0.848$
Bayesian information criteria	1211.3	1233.5	1290.6

SOFA - Sequential Organ Failure Assessment; ECOG - Eastern Cooperative Oncology Group; AMIB - *Associação de Medicina Intensiva Brasileira*; NA - not aplicable.

Table 6S - Logistic regression model results of the White score and AMIB score compared to a more flexible score incorporating all variables among patients older than 50 years

Variable	Model 1 (Spline age + SOFA continuous + White + ECOG AMIB) OR (95%CI)	Model 2 (White score) OR (95%CI)	Model 3 (AMIB score) OR (95%CI)
Non-linear spline function of age	Spline ($p < 0.001$)	NA	NA
Age (years)			
50 - 60	NA	Reference	NA
61 - 74	NA	1.42 (0.98 - 2.06); p = 0.065	NA
≥ 75	NA	4.14 (2.4 - 7.14); $p < 0.001$	NA
SOFA			
1 point	1.2 (1.2 - 1.3); $p < 0.001$	NA	NA
1st level (AMIB 0 - 8; White 0 - 5)	NA	Reference	Reference
2nd level (AMIB 9 - 13*; White 6 - 9)	NA	1.97 (1.35 - 2.88); $p < 0.001$	2.19 (1.5 - 3.21); $p < 0.001$
3rd level (AMIB 14 - 24; White 10 - 12)	NA	3.85 (2.36 - 6.30); $p < 0.001$	5.95 (2.62 - 13.48); $p < 0.001$
4th level (White 13 - 24)	NA	13.95 (3.18 - 61.21); $p < 0.001$	NA
Comorbidities			
Minor	0.81 (0.5 - 1.3); p = 0.375	0.90 (0.57 - 1.41); p = 0.637	NA
Major	1.1 (0.63 - 1.93); p = 0.737	1.20 (0.69 - 2.10); p = 0.523	1.66 (1.13 - 2.43); p = 0.009
Severe	2.12 (0.87 - 5.13); p = 0.097	2.68 (1.23 - 5.85); p = 0.013	NA
ECOG categories			
0 - 1	Reference	NA	Reference
2	1.06 (0.6 - 1.8); p = 0.85	NA	1.64 (1.01 - 2.66); p = 0.045
3	1.43 (0.56 - 3.66); p = 0.454	NA	2.03 (0.89 - 4.65); p = 0.093
4	0.43 (0.37 - 5.11); p = 0.506	NA	0.99 (0.09 - 11.02); p = 0.998
Bayesian information criteria	853.1	856.3	883.2

SOFA - Sequential Organ Failure Assessment; ECOG - Eastern Cooperative Oncology Group; AMIB - *Associação de Medicina Intensiva Brasileira*; NA - not applicable. * AMIB´s criteria categories 2 and 3 were merged due to sparse data in the former category.

Table 7S - Discrimination, accuracy (Brier score), and calibration of the proposed models after imputation of missing data for patients below 50 years of age

Model	AUROC (95%Cis*)	Brier score (95%CIs†)
AMIB criteria	0.66 (0.63 - 0.70)	0.22 (0.21 - 0.23)
White criteria	0.72 (0.69 - 0.75)	0.21 (0.20 - 0.22)

AUROC - area under the Receiver Operating Characteristic curve; AMIB - *Associação de Medicina Intensiva Brasileira*. * Asymptotic 95% confidence intervals; † Bootstrapped 95% confidence intervals (N = 200).

For this imputation model, we imputed Karnofsky Performance Status and Clinical Frailty Scale and then derived Eastern Cooperative Oncology Group and comorbidity severity as passive variables. We included age, sex, components of the Sequential Organ Failure Assessment score, comorbidities, and the outcome, hospital mortality, in the imputation model, following multiple imputation recommendations.[1] We did not include auxiliary variables. The imputation model was predictive mean matching with 30 imputations to account for the proportion of missing data. We then performed the same predictive performance analysis on the derived AMIB and White scores to compare them.

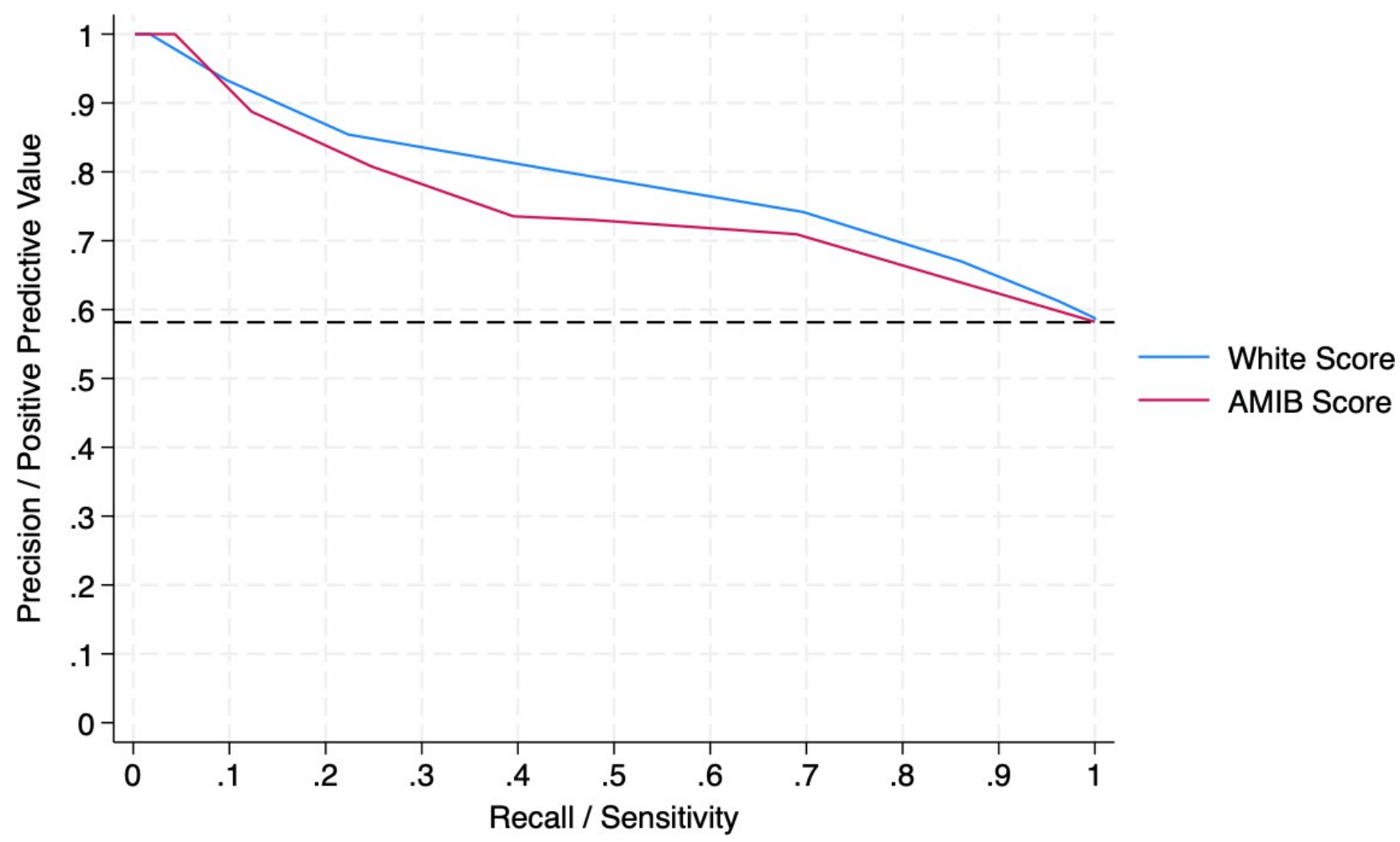

AMIB - *Associação de Medicina Intensiva Brasileira*.

Figure 1S - Precision-recall curve for the comparison of AMIB and White scores.

The dotted line represents the sample's hospital mortality.

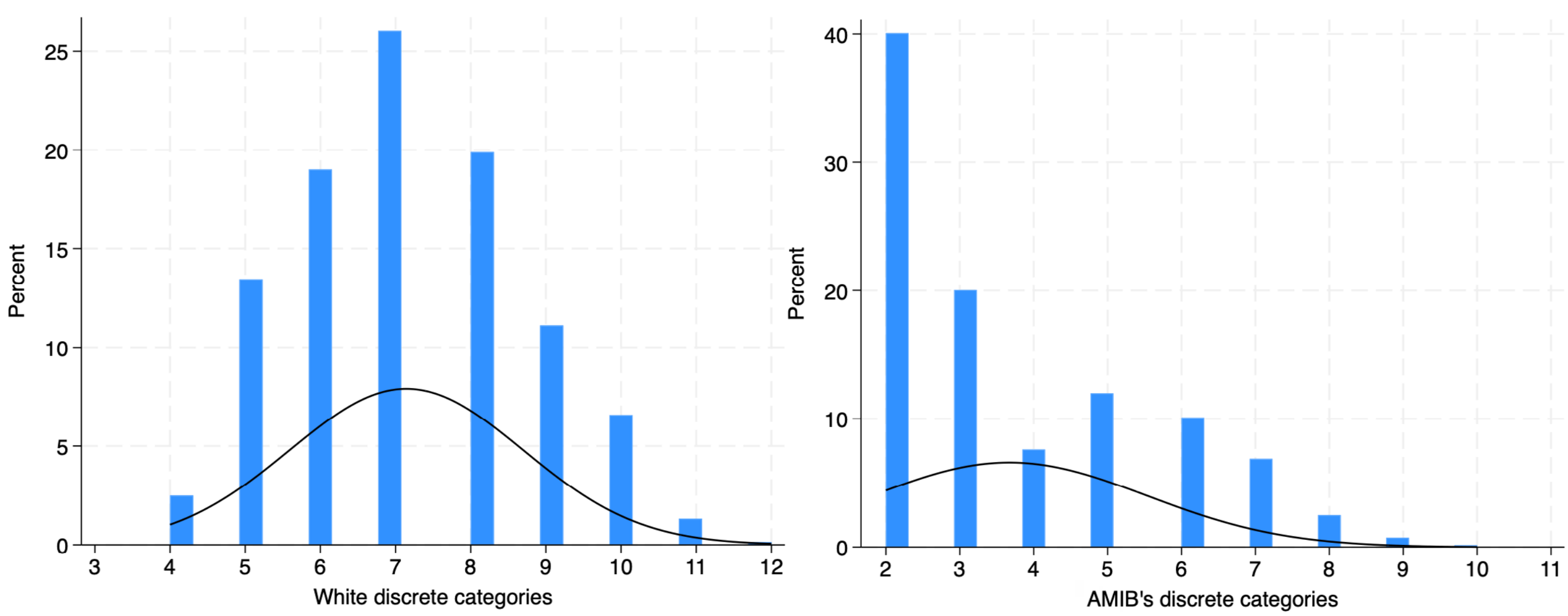

AMIB - *Associação de Medicina Intensiva Brasileira*.

Figure 2S - Distribution of White's and AMIB's criteria discrete categories among patients older than 50 years.

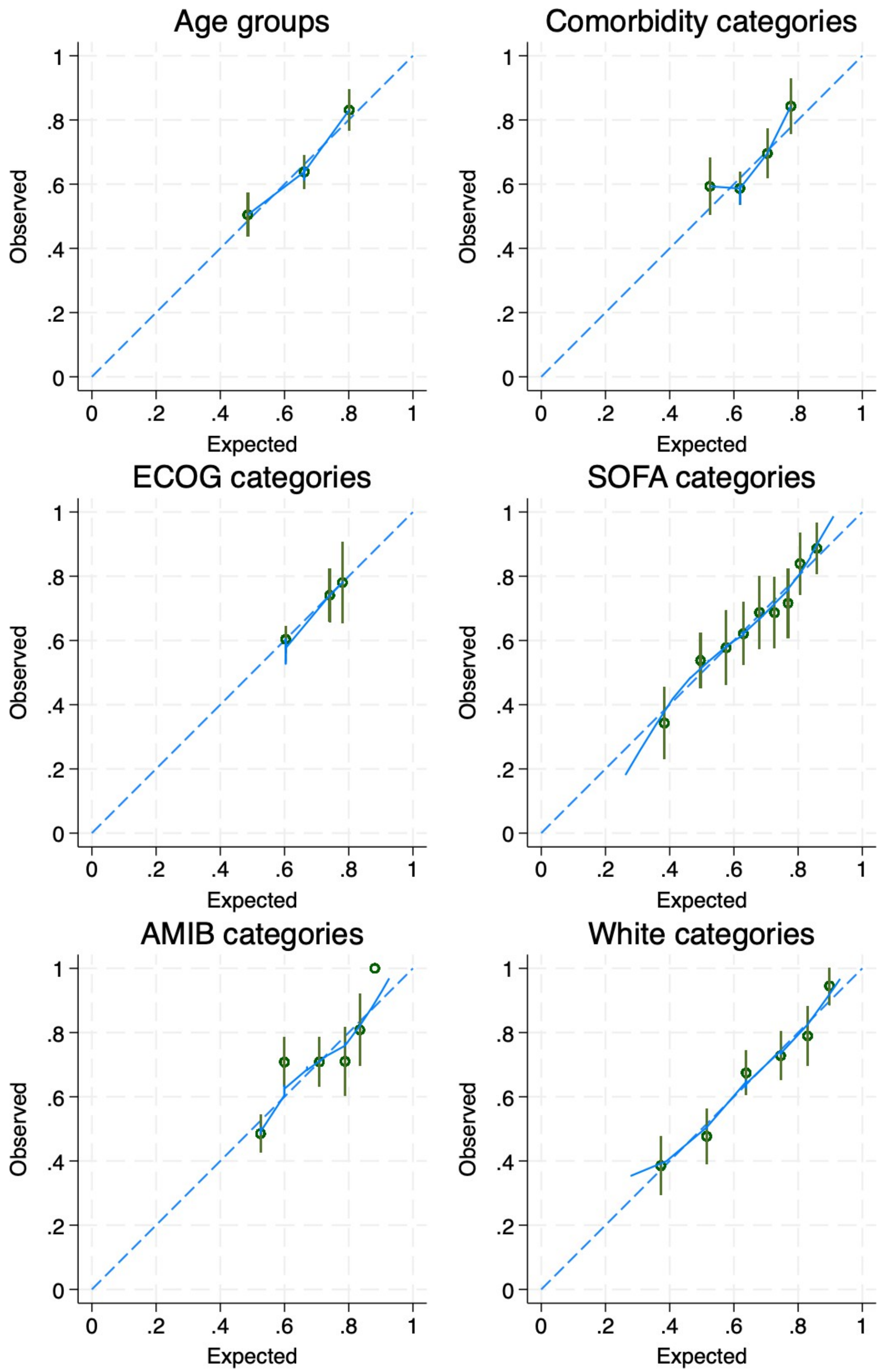

ECOG - Eastern Cooperative Oncology Group; SOFA - Sequential Organ Failure Assessment; AMIB - *Associação de Medicina Intensiva Brasileira*.

Figure 3S - Calibration plots among patients older than 50 years.

These calibration plots demonstrate White's criteria and AMIB's criteria LOWESS (Locally Weighted Scatterplot Smoothing) curves (blue lines), along with their discrete categories with 95% confidence intervals (green). Age groups, comorbidities, Eastern Cooperative Oncology Group, and Sequential Organ Failure Assessment categories are also presented independently as they are components of either White's or AMIB's criteria.

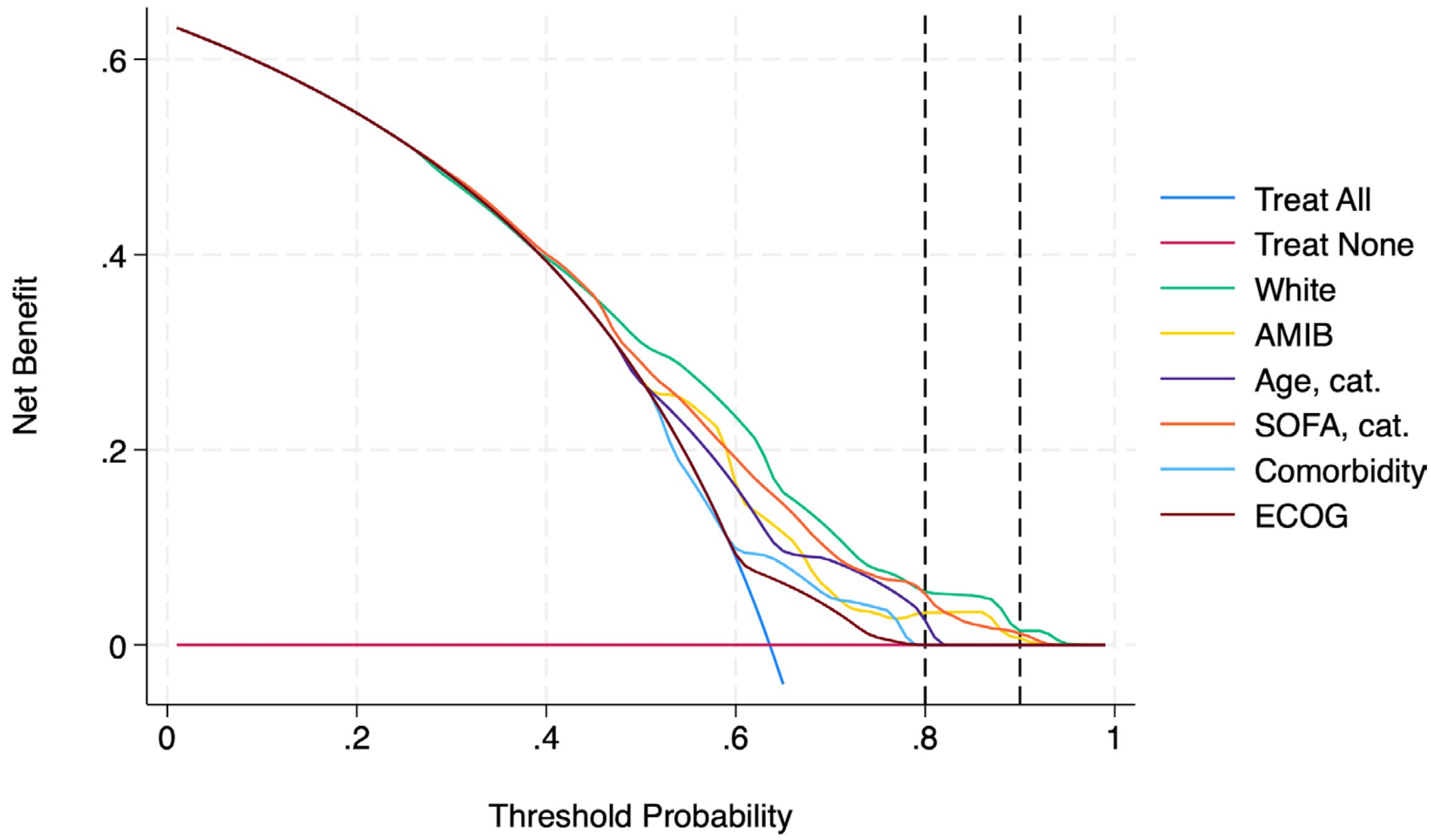

AMIB - *Associação de Medicina Intensiva Brasileira*; ECOG - Eastern Cooperative Oncology Group; SOFA - Sequential Organ Failure Assessment.

Figure 4S - Decision curve analysis plots among patients older than 50 years.

The decision curve analysis plot presents the net benefit of adopting each model across different thresholds for hospital mortality probability to inform individual decisions (e.g., intensive care unit triage). The x-axis point where the "Treat all" strategy meets the "Treat none" strategy represents the cohort observed hospital mortality. The vertical dashed lines represent the 80% and 90% probability thresholds, which could be considered thresholds for identifying patients less likely to benefit from intensive care unit care. This figure demonstrates that, although the White model has higher net benefit across intermediate threshold probabilities (where no decision is likely to be made), no model has meaningful net benefit beyond 80% or 90% thresholds.

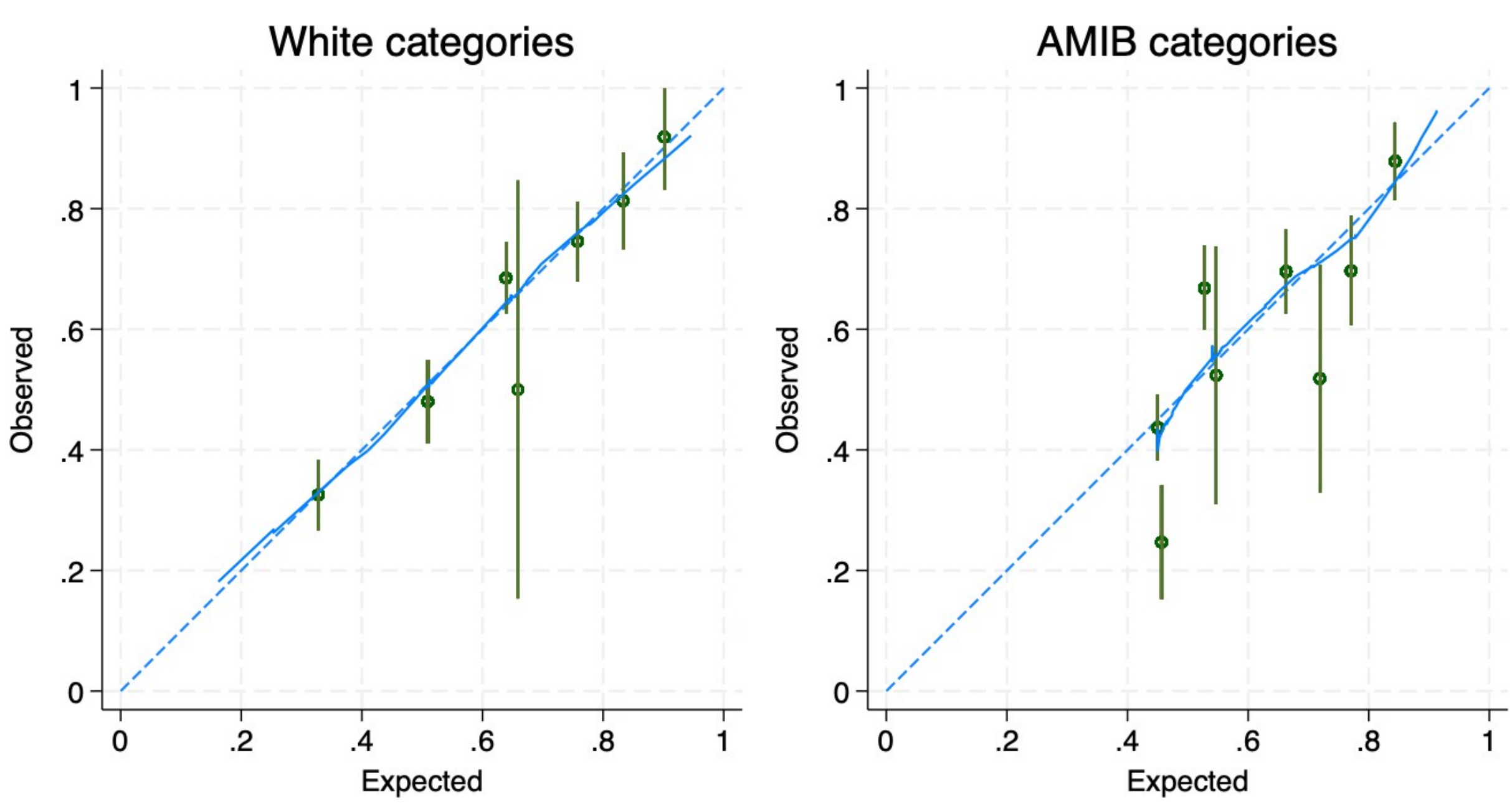

AMIB - *Associação de Medicina Intensiva Brasileira*.

Figure 5S - Calibration plots after imputation of missing data for patients below 50 years of age.

These calibration plots demonstrate White's and AMIB's criteria LOWESS (Locally Weighted Scatterplot Smoothing) curves (blue lines), along with their discrete categories with 95% confidence intervals (green) after imputation for missing data among patients younger than 50 years.

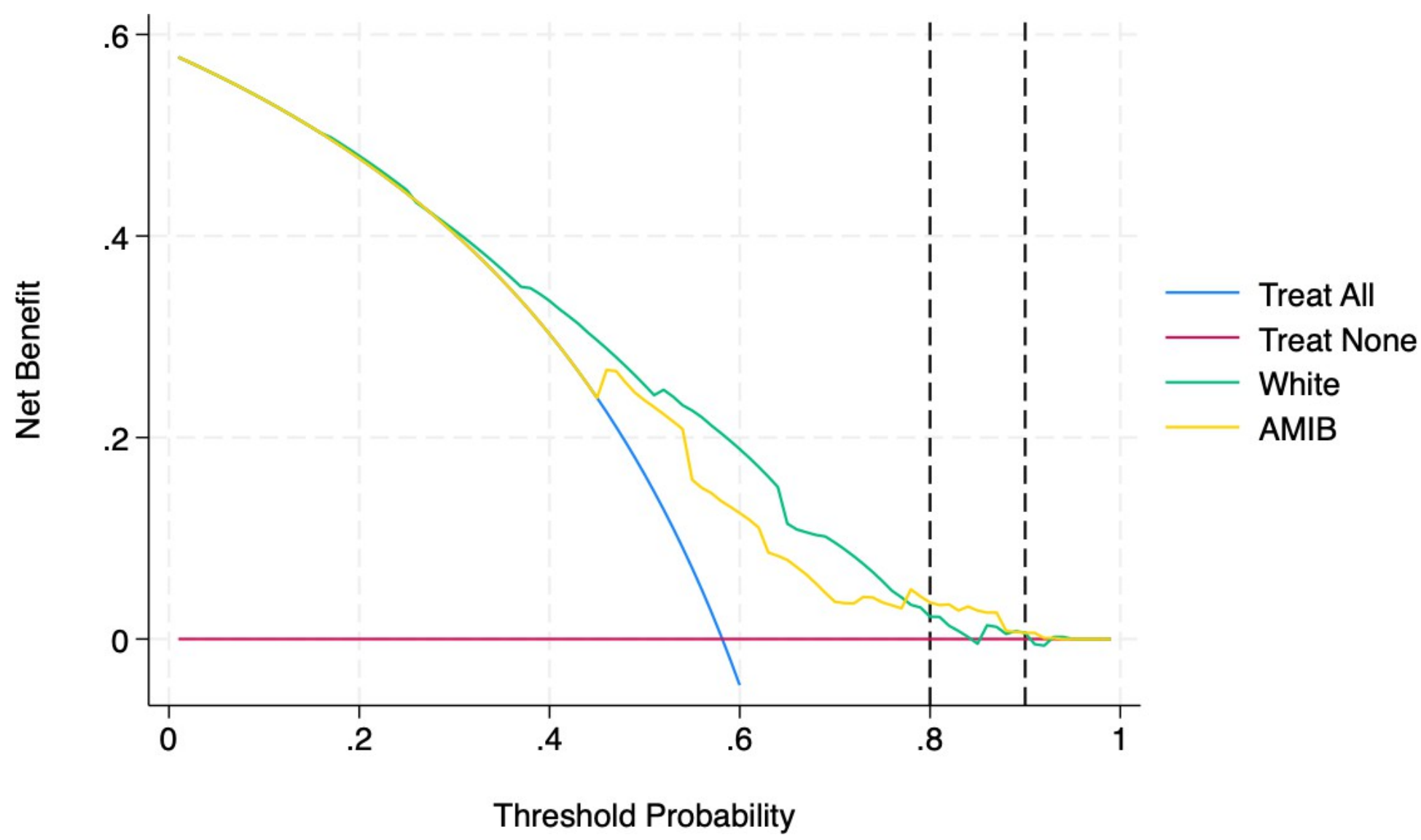

AMIB - *Associação de Medicina Intensiva Brasileira*.

Figure 6S - Decision curve analysis plots after imputation of missing data for patients below 50 years of age.

REFERENCE

1. Sterne JA, White IR, Carlin JB, Spratt M, Royston P, Kenward MG, et al. Multiple imputation for missing data in epidemiological and clinical research: potential and pitfalls. BMJ. 2009;338:b2393.
